# Revised recommendations concerning palivizumab prophylaxis for respiratory syncytial virus (RSV)

**DOI:** 10.1186/s13052-015-0203-x

**Published:** 2015-12-15

**Authors:** Lina Bollani, Eugenio Baraldi, Gaetano Chirico, Andrea Dotta, Marcello Lanari, Antonello Del Vecchio, Paolo Manzoni, Antonio Boldrini, Piermichele Paolillo, Sandra Di Fabio, Luigi Orfeo, Mauro Stronati, Costantino Romagnoli, on behalf of the Italian Society of Neonatology

**Affiliations:** Unità di Neonatologia, Patologia Neonatale e Terapia Intensiva, Policlinico S. Matteo, Pavia, Italy; U.O.C. Terapia Intensiva e patologia neonatale, A.O. Padova, Padova, Italy; U.O. C. Neonatologia e Terapia Intensiva neonatale, A.O. Spedali civili, Brescia, Italy; U.O.C. Terapia intensiva neonatale, Ospedale pediatrico Bambino Gesù, Roma, Italy; U.O. Pediatria E Neonatologia , Ospedale Nuovo S. Maria della Scaletta, Imola, Italy; U.O. Utin e Neonatologia, Azienda Ospedaliera di Venere e Giovanni XXIII, Bari, Italy; U.O. Neonatologia e TIN ospedaliera, Azienda Ospedaliera OIRM-S. Anna, Torino, Italy; U.O. Nido, Patologia Neonatale e Terapia Intensiva Neonatale, Ospedale Santa Chiara, Pisa, Italy; U.O. Divisione di Neonatologia, Policlinico Caslino, Roma, Italy; U.O. Neonatologia, Presidio Ospedaliero S. Salvatore, L’Aquila, Italy; U.O. Nido, Patologia Neonatale e Terapia Intensiva Neonatale, Azienda Ospedaliera G. Rummo, Benevento, Italy; Unità di Neonatologia, Patologia Neonatale e Terapia Intensiva, Ospedale Policlinico S. Matteo, Pavia, Italy; U.O.C. Neonatologia, Policlinico Universitario A. Gemelli, Roma, Italy; Division of Neonatology, Department of Pediatrics, Catholic University S.H., Largo A. Gemelli,8, Rome, 00168 Italy

## Abstract

Respiratory Syncytial Virus infections are one of the leading causes of severe respiratory diseases that require hospitalization and, in some cases, intensive care. Once resolved, there may be respiratory sequelae of varying severity. The lack of effective treatments for bronchiolitis and the lack of vaccines for RSV accentuate the role of prevention in decreasing the impact of this disease.

Prevention of bronchiolitis strongly relies on the adoption of environment and the hygienic behavior measures; an additional prophylactic effect may be offered, in selected cases, by Palivizumab, a humanized monoclonal antibody produced by recombinant DNA technology, able to prevent RSV infection by blocking viral replication.

After many years the Italian Society of Neonatology, on the basis of the most recent scientific knowledge, has decided to revise recommendations for the use of palivizumab in the prevention of RSV infection.

## Background

Respiratory Syncytial Virus infections are one of the leading causes of severe respiratory diseases that require hospitalization and, in some cases, intensive care. Once resolved, there may be respiratory sequelae of varying severity.

## Elements of virology

The respiratory syncytial virus (RSV) was isolated for the first time in 1955 in a monkey. In man, the virus was described in 1957 in two neonates presenting with an airway infection [1].

It belongs to the order *Monegavirales*, family *Paramyxoviridae*, subfamily *Pneumovirinae*, genus *Pneumovirus*.

The RSV virion consists of a helical symmetrical nucleocapsid surrounded by a lipid envelope, normally derived from the host cell, and it contains three transmembrane glycoproteins shaped like short spikes on its surface. Although glycoprotein G is in charge of mediating adhesion to the ciliated epithelium of the airways and entry of RSV in the infected cell, it is not strictly necessary nor sufficient to cause the disease. There are two antigenic subgroups of RSV, A and B, which may be identified based on the different conformation of glycoprotein G. Fusion F protein instead maintains its sequence in the two subgroups and plays the crucial role of allowing viral penetration in the cells via fusion of the viral envelope with the cytoplasmic membrane. The third protein is a small hydrophobic protein called SH, and is a viroporin capable of modifying cell membrane permeability [2]. Once RSV has penetrated in the host cell (mediated by glycoproteins G and F) viral genome transcription and viral replication take place in the cytoplasm, where proteins and viral RNA accumulate and peak 15–20 hours after infection. At this point, the viral progeny may start to be released from the cell and continue for approximately 48 hours, or until the cell has been completely destroyed. This latter phase might be preceded by the development of cell syncytia (major cytopathogenic effect of the virus) [2, 3].

## Epidemiology, clinical aspects, long-term complications

RSV is the most frequent cause of airway infections in children under the age of 2 years , and bronchiolitis is the main cause for hospitalization during the first year of life (approximately 1 % of children in Europe and the United States), with peak of hospitalization at 2 months of age [4]. Children younger than 3 months or who present with pre-existing risk factors (prematurity, bronchopulmonary dysplasia, congenital heart diseases, immunodeficiency, neuromuscular diseases) are especially at risk for severe disease and hospitalization, sometime with the need for admission to the intensive care unit. In industrialized countries, bronchiolitis, caused by a viral infection during the first year of life, continues to remain an important cause of death [5]. In Italy, the epidemic season is between November and March, with a peak in January – February, as shown by Italian epidemiological studies [6].

The diagnosis of bronchiolitis is based on clinical criteria: rhinorrhea and/or upper airway infection, a first episode of respiratory distress with crackles and/or wheezing, polypnea, use of accessory muscle and chest retractions, difficulties in taking fluids and food, hypoxia [7–9]. Children with acute bronchiolitis may present with a wide range of clinical presentations that range from mild respiratory distress to impending respiratory failure. The immune response to the RSV infection in children who develop bronchiolitis is characterized by the presence of a major neutrophil-mediated inflammation of the airways.

Hospitalization in case of bronchiolitis is indicated in presence of hypoxia (O_2_ saturation <90-92 % at ambient air), moderate to severe respiratory distress, dehydration, apnea. Other criteria to be taken into account are gestational age as well as postnatal age, belonging to categories at risk, abnormal state of consciousness and responsiveness, decreased fluid intake (<50 % of habitual intake), unfavorable social and environmental factors. Neonates or infants with severe bronchiolitis should be admitted to a Pediatric Intensive Care Unit if presenting with respiratory failure, and severe impairment of general conditions [9].

There is no evidence of efficacy for many of the therapies commonly used to treat bronchiolitis (bronchodilators, steroids, antibiotics) [7] and supportive treatment (warm humidified oxygen, high flows and hydration) still remains the approach recommended by the leading international and national guidelines [7–9]. Recent studies suggest that nebulization of 3 % hypertonic saline solution provides clinical benefit, however data in this regard are conflicting [10–13]. Nebulized epinephrine might be useful in the hospital setting, as a rescue agent in severe disease [8]. The lack of effective treatments for bronchiolitis and the lack of vaccines for RSV accentuate the role of prevention in decreasing the impact of this disease.

Infection can be consistently controlled by the application of environmental, hygienic and sanitary measures to cut diffusion of the virus to a minimum and on pharmacological prophylaxis with administration of palivizumab during the epidemic season to children at high risk [14, 15]. Environmental prophylaxis, especially during the epidemic season, is essential to decrease transmission of RSV in the hospital and outpatient clinic settings, since it is easily spread through the air carried by saliva droplets and via contact with contaminated objects and surfaces (hands, clothing, toys, medical instruments, kitchen utensils, etc.) on which the virus may deposit and remain active for several hours. Environmental prophylaxis is performed by frequent washing and decontamination of hands using alcohol-based solutions, and by cleaning solid surfaces with water and disinfectants (alcohol-based detergents are preferable). In the hospital or outpatient clinic setting, multiple-use medical equipment (e.g., stethoscope) must also be decontaminated. The use of disposable gloves and white coats by healthcare operators who have contact with the patient is recommended as well. Thanks to compliance with these recommendations, hospital–acquired RSV infections have decreased by 39–50 % [16]. It is very important that breast-feeding be recommended as it decreases infectious respiratory morbidity [7, 9, 17]. Parent education is furthermore essential to avoid exposure of children to secondhand cigarette smoke, which increases the risk of developing bronchiolitis and respiratory infections [7]. Also preventing exposure of the fetus to maternal secondhand smoke during pregnancy is of especially importance [7, 14, 15, 18].

## Later respiratory outcomes of bronchiolitis

An RSV infection in infants, in particular in those who required hospitalization, might interfere with the normal development of the immune system and of the lungs, and might be associated with an increased incidence of recurrent bronchospasm in preschoolers, asthma, and with decreased respiratory function in school-aged children [19, 20].

Recent follow-up studies conducted through adulthood (18 and 30 years of age) have demonstrated that up to 30-40 % of subjects who had been previously hospitalized because of bronchiolitis suffer from asthma and use anti-asthma drugs [21].

A recent randomized, double-blind, placebo-controlled study demonstrated that prophylaxis with palivizumab given to healthy preterm babies (33 – 35 weeks gestational age) is able to decrease by 61 % the number of days with bronchospasm throughout the first year of life, thus supporting the hypothesis of a direct damage caused by the virus. No similar studies are available in full-term children [22].

## Scope of the recommendations

The Recommendations drafted by the Italian Society of Neonatology concerning palivizumab prophylaxis for respiratory syncytial virus infection intends to provide a shared operating guidelines, for a uniform prescription on the basis of the most recent scientific knowledge. The levels of evidence are indicated in the Table 1.

**Table 1:** Level of evidence and strength of recommendation for RSV prophylaxis with palivizumab

	Level of evidence
I	evidence obtained by randomized controlled clinical studies and/or by systematic reviews of randomized studies
II	evidence obtained from an individual and adequately designed randomized study
III	evidence obtained by cohort studies with concurrent or historical controls, or a meta-analysis thereof.
IV	evidence obtained by retrospective, case-controlled studies or meta-analysis
V	evidence obtained from case-series studies without a control group
VI	evidence based on the opinion of authoritative experts or of committees of experts as indicated in guidelines or a consensus conference, or based on the opinion of the members of the workgroup responsible for this guideline
	Strength of recommendations
A	the performance of that particular procedure or diagnostic test is strongly recommended (it indicates a particular recommendation supported by good quality albeit not necessarily type I or II scientific evidence)
B	there is some doubt that the procedure/intervention must be always recommended, but it is thought that its execution has to attentively be considered
C	there is substantial uncertainty in favor of or against the recommendation to perform the procedure or the intervention
D	performing the procedure is not recommended
E	performing the procedure is strongly discouraged

## Environmental hygiene prevention

Strict adherence to infection-control practices is important to reduce the spread of RSV disease and can be affective in containing respiratory virus epidemics or in hospital wards: isolation, cohorting, social distancing, restricting visitation by young children, barriers, personal protection, surgical masks, handwashing [23]. Encouraging breast-feeding throughout the baby’s first year of life, and avoiding exposure to secondhand smoke are effective methods to decrease prevalence of the RSV disease in the pediatric population.

**[Level of evidence II – strength of recommendation A]**

## Palivizumab

Palivizumab (Synagis; Medimmune, Inc., Gaithersburg, MD) [24] is a humanized monoclonal antibody (IgG1) produced by recombinant DNA technology, directed against an epitope in the A antigenic site of the fusion F protein of RSV. Palivizumab acts by inhibiting and neutralizing the fusion action of the F protein, thus blocking viral replication.

The multicenter, randomized, double-blind, controlled clinical study IMpact (RSV-IMpact trial) licensed in 1998 used palivizumab at the dosage of 15 mg/kg administered via intramuscular injection once a month, during the epidemic season, for a total of 5 administrations [25]. Despite it being widely used for more than a decade, only a study recently performed by Robbie et al. [26] analyzed the various pharmacokinetic scenarios for palivizumab in infants, children and adults with several clinical presentations. The few previously conducted pharmacokinetic studies only described mean serum concentrations and half-life ranging between 17 [27] and 26.8 days [28]. The manufacturing company itself, in 2012, indicated a marked inter-individual variability in serum concentrations of palivizumab when administered as per the registered regimen [24].

The study performed by Robbie et al. [26] also assessed the impact on inter-individual variability of the formation of antidrug antibodies (ADA) and of a number of specific pathological conditions, to verify if an adequate protection was ensured with only 3 administrations instead of the contemplated 5 doses. The conclusions of the study performed by Robbie highlighted a slight difference in the pharmacokinetic profile between full-term and preterm neonates, and showed that 5 administrations during the epidemic season guarantee greater and longer lasting protection compared to the scheme with only 3 seasonal administrations. These findings were confirmed by subsequent studies [29]. Therefore, the scheme which contemplates a monthly intramuscular administration at the dosage of 15 mg/kg for 5 months, as indicated in the registered scheme, would appear to be more appropriate.

**[Level of evidence II – strength of recommendation A]**

## Palivizumab prophylaxis in preterm infants

The efficacy and safety profile of palivizumab was proved by a multicenter, randomized, double-blind, controlled clinical trial (RSV-IMpact trial) [25].

1502 preterm babies of ≤ 35 weeks gestational age (GA) and ≤ 6 months of age at the beginning of the epidemic season, or preterm babies suffering from bronchopulmonary dysplasia (BPD) ≤ 2 years of age at the beginning of the epidemic season and requiring specific medical therapy (oxygen support, bronchodilators, diuretics, corticosteroids) in the 6 months preceding the beginning of the epidemic season, were randomized to receive palivizumab by intramuscular injection at the dose of 15 mg/kg once a month for 5 months or placebo.

The results obtained showed a statistically significant decrease by 55 % (from 10.6 to 4.8 %) in RSV-related hospitalizations in subjects who received palivizumab (*n* = 500) compared to subjects who received placebo (*n* = 1002).

In the same year, the American Academy of Pediatrics (AAP) [30] recommended the use of palivizumab for prophylaxis of the RSV infection in preterm babies of ≤ 35 weeks GA and in preterm babies presenting with BPD.

Subsequent modifications of the AAP recommendations concerning the use of palivizumab [7, 14, 15, 31] took into account the new epidemiological data available concerning the incidence of hospitalizations due to bronchiolitis in the United States, the impact of gestational age, pharmacokinetics of palivizumab and other risk factors observed in the United States. In particular, the AAP cited a recent prospective study [32] which demonstrated a mean incidence of hospitalizations caused by RSV-induced bronchiolitis which was significantly greater in neonates < 30 weeks GA compared to more mature preterm babies.

It must be pointed out that other data available in literature do not agree with this report, citing varying gestational age cut off values as the decisive elements underlying higher and lower risks of hospitalizations in preterm neonates [33, 34].

For this reason, it is difficult to establish a gestational age “threshold” differentiating between a high risk and a lower risk in preterm neonates of less than 32 weeks GA. It is undeniable that risks of RVS-related hospitalization decrease as gestational age increases, but such decrease occurs progressively and not on a step-by-step basis. All this appears to be consistent with the position of the Cochrane Review that, when discussing the efficacy of palivizumab in preventing RSV Hospitalization, generically states “… evidence that palivizumab is effective in reducing incidence of severe lower respiratory tract infection in neonates suffering from CLD or CHD and in preterm babies”, and does not touch upon gestational age subgroups but only uses the generic definition of “preterm” [35].

A pragmatic approach might in any case take into account the coexistence of specific risk factors alongside gestational age alone.

A recent paper by Lanari et al. [36] concerning risk factors for RSV hospitalization and involving 2314 neonates, demonstrated that neonates of 33–34 weeks GA have a twofold risk of hospitalization caused by bronchiolitis infection (83 % caused by RSV) compared to full-term babies. Moreover, the risk for neonates of 35– 37 weeks GA is one and a half times that of full-term children. These data are confirmed by other international studies suggesting the lack of difference in terms of risk of hospitalization because of severe RSV infection between these subjects and children with a lower gestational age [34, 37–39].

It must be also noted that the Italian study states that for the majority of hospitalizations because of RVS infection occurred during the first six months of life [36].

Lacking any clear scientific evidence, neonates of 29–35 weeks GA may receive prophylaxis, limited to the first 6 months of life, in the presence of risk factors for severe RSV infection. The risk factors identified by various studies are: male gender, exposure to maternal smoke and secondhand smoke, treatment with surfactant, living with siblings aged <10 years, attendance at daycare and births occurring close to or during the epidemic season [39–45]. All these risk factors were noted in the population of neonates of 33–35 weeks GA of the study carried out in Italy [36].

**For infants of gestational age <29 weeks and age ≤12 months at the beginning of the epidemic season: LEVEL OF EVIDENCE II – Strength of recommendation A.**

**For infants of 29–35 weeks gestational age and age ≤6 months at the beginning of the epidemic season, prophylaxis with Palivizumab might be taken into consideration in presence of risk conditions predisposing to severe infections and/or need for hospitalization.**

**[LEVEL OF EVIDENCE IV – Strength of recommendation B]**

There are no data supporting the use of palivizumab to control hospital epidemics in the Neonatal Intensive Care Unit or in Neonatology wards [14, 15].

## Palivizumab prophylaxis in children with Bronchopulmonary Dysplasia (BPD)

Bronchopulmonary dysplasia (BPD) is one of the most significant sequelae associated with preterm births. This definition describes the chronic lung disease that follows a neonatal respiratory disorder [46]. BPD is defined as the need for O_2_ therapy for at least 28 days after birth, and severity is defined based on the need for respiratory support required at 36 weeks post menstrual age [47]. There is substantial consensus concerning treatment with Palivizumab in infants diagnosed with BPD during their first year of life [9, 14, 15]. A recent Cochrane Collaboration review, that included studies on BPD patients, concluded that there is evidence that prophylaxis with Palivizumab is effective in decreasing frequency of hospitalizations caused by RSV infections in these patients [35].

Throughout the second year of life, prophylaxis with Palivizumab is recommended for children diagnosed with BPD who require medical therapy (oxygen, bronchodilators, diuretics or chronic steroids) in the six months preceding the beginning of the epidemic season [9, 14, 15].

**[LEVEL OF EVIDENCE II – Strength of recommendation A]**

## Palivizumab prophylaxis in children with heart diseases

In Italy, approximately 8 neonates out of 1000 suffer from congenital heart disease.

In 1982, MacDonald et al. reported for the first time that children with congenital heart disease are at increased risk of severe lower airway respiratory diseases associated with RVS infections [48].

Subsequent studies confirm that these children present an increased risk of hospitalization, with longer hospital stays, admission to the intensive care unit, and frequent need for oxygen therapy and mechanical ventilation for longer periods compared to children without any risk factors [49–53].

Cumulative data from recent published reviews, confirmed an increased mortality rate within patients with significant congenital heart disease (CHD) associated with RSV, even if mortality seems to be in marked decline through years [54, 55].

Moreover, the higher risk of hospital-acquired infection caused by RVS is an unfavorable perioperative prognostic factor in heart diseases with surgical indication.

In 2003 Feltes et al. [56] published data generated by a multicenter, prospective, placebo-controlled study of the administration of palivizumab in a population of infants aged less than 24 months, suffering from hemodynamically significant heart disease. The study enrolled 1287 patients and the results obtained showed a significant 4.4 % decrease of the hospitalization rate (9.7 % in patients who received placebo compared to 5.3 % in patients who received palivizumab, *p* = 0.003) and a decrease in morbidity and in need of intensity of care. Prophylaxis with palivizumab appeared to be effective also in patients with non-cyanogenic heart disease. No case of adverse effects of prophylaxis on surgical outcome was noted.

Based on the currently available data in literature, it may be considered useful to recommend palivizumab prophylaxis for children with hemodynamically significant congenital heart disease, younger than 12 months at the beginning of the epidemic season, and who meet the following criteria:

- Infants with cyanogenic heart disease prior to the surgical procedure or after a palliative procedure, on indication of the pediatric cardiologist based on the hemodynamic status of the patient. **[LEVEL OF EVIDENCE IV – Strength of recommendation A]**
- infants with non-cyanogenic heart disease on therapy for congestive heart failure and who are scheduled to undergo surgery; **[LEVEL OF EVIDENCE II – Strength of recommendation A]**
- infants with moderate to severe pulmonary hypertension; **[LEVEL OF EVIDENCE II – Strength of recommendation A]**
- Infants with surgically repaired congenital heart disease who continue to need therapy for congestive heart failure; **[LEVEL OF EVIDENCE II – Strength of recommendation A]**
- infants suffering from congestive cardiomyopathy on treatment with anti-congestive medication; **[LEVEL OF EVIDENCE II – Strength of recommendation A]**
- infants on the waiting list for heart transplantation or in the post-transplantation period. **[LEVEL OF EVIDENCE II – Strength of recommendation A]**

Palivizumab prophylaxis is not required in patients with non-hemodynamically significant congenital heart disease (for instance: ostium secundum atrial septal defect, small ventricular septal defect, pulmonary artery stenosis, uncomplicated aortic artery stenosis, mild aortic coarctation, patent ductus arteriosus).

The current indications proposed by the Italian Society of Pediatric Cardiology (SIPC) are summarized in the intersociety document [9].

In consideration of a 58 % decrease in mean palivizumab concentrations observed in children who undergo surgical procedures with need for cardiopulmonary bypass graft during prophylaxis, administration of an additional dose of palivizumab after surgery is recommended [32].

Children who underwent heart transplantation during the epidemic season might benefit from palivizumab prophylaxis also during their second year of life [14, 15].

## Palivizumab prophylaxis in children belonging to special categories: cystic fibrosis, Down syndrome, congenital diaphragmatic hernia, neuromuscular diseases, immunodeficiency, accumulation disorder, esophageal atresia, lung transplantation

The Italian intersociety document and the most recent technical report issued by the AAP concerning indications to palivizumab prophylaxis, concur that for these categories of patients, a conclusive determination of the risk of severe RSV infection and of the possible worsening of the underlying disease represent a true dilemma [9, 14]. Indeed, as many of these diseases are rare, it is not feasible to conduct randomized controlled studies both on the actual risk of severe RSV disease and on the effectiveness of prophylaxis with palivizumab.

- **Lung malformations or neuromuscular diseases:** in children with neuromuscular diseases or anatomical abnormalities of the lungs with impaired lower airway clearance, pulmonary malformations, tracheoesophageal fistula, severe upper airway dysfunction or tracheostomy, palivizumab prophylaxis might be taken into consideration during the first year of life [57–62].
- **Patients with immunodeficiency:** No population-based epidemiological data are available concerning incidence or severity of RSV infection in patients undergoing solid organ or hematopoietic cell line transplantation, or in patients undergoing chemotherapy or presenting with immunodeficiency because of other causes. Despite retrospective studies and evaluation of clinical risks associated with immunodeficiency, there is no evidence of a true advantage in using palivizumab, and further research is warranted to reach a robust conclusion concerning indication for prophylaxis [63].
- **Down Syndrome patients:** The existing historic cohort or birth cohort studies underscore a high risk of RSV-related hospitalization in Down children, regardless of the presence of any concomitant heart disease. Therefore, despite the lack of randomized controlled studies and of robust evidence generated by prospective studies, it is legitimate to believe that patients with Down syndrome might be considered to be at greater risk of developing a severe form of RSV-related bronchiolitis. Therefore, it is reasonable to take into consideration recourse to palivizumab prophylaxis in these patients, provided their clinical conditions are taken into account [64–67].
- **Patients with cystic fibrosis:** several studies have demonstrated a higher risk of RSV infection-related hospitalization in patients who suffer from cystic fibrosis [68, 69]. A study conducted by Winterstein et al. [70] demonstrated a decreasing trend of RSV infection hospitalizations in patients receiving prophylaxis with palivizumab, as was already shown by the Delphy study [71].

Despite the lack of robust scientific evidence, the expert opinion is that patients suffering from the most severe forms of diseases listed above might benefit from prophylaxis with palivizumab during the epidemic season [8, 72, 73].

**[LEVEL OF EVIDENCE V - Strength of recommendation B]**

## Conclusions

The conclusions of these recommendations are summarized in Table 2.

**Table 2:** Recommendation summary table

	Level of evidence	Strength of recommendation
Environmental hygiene prevention	II	A
Efficacy and safety of palivizumab prevention	II	A
Dose of 15 mg/Kg once a month for 5 months	II	A
Prophylaxis in subjects with <29 weeks GA and aged ≤ 12 months at the beginning of epidemic season	II	A
Prophylaxis in subjects with 29–35 weeks GA and aged ≤ 6 months at the beginning of epidemic season	IV	B
Palivizumab prophylaxis in infants with bronchopulmonary dysplasia and aged ≤ 12 months at the beginning of epidemic season, and during the second year of life in children who require medical therapy	II	A
Prophylaxis in infants with severe congenital heart disease and aged ≤ 12 months at the beginning of epidemic season	II	A
Prophylaxis in infants with cystic fibrosis, Down syndrome, congenital diaphragmatic hernia, neuromuscular diseases, immunodeficiency, accumulation disorders, esophageal atresia, lung transplantation	V	B

Italian Society of Neonatology hopes that this document will be useful for all neonatologists and pediatricians involved in the management of infants at risk of RSV infection, and suggests that these recommendations should be revised almost every 3–5 years on the basis of future researches.

These recommendations are been compiled for being used in the Italian population and they are based on Italians epidemiological data and on other inter-society documents.

## Abbreviations

BPD: bronchopulmonary dysplasia
CHD: congenital heart disease
CLD: chronic lung disease
CPAP: continuous positive airway pressure
GA: gestational age
RSV: respiratory syncithial virus
